# Mulberry Leaf Supplements Effecting Anti-Inflammatory Genes and Improving Obesity in Elderly Overweight Dogs

**DOI:** 10.3390/ijms232315215

**Published:** 2022-12-02

**Authors:** Miey Park, Varun Jaiswal, Kihyun Kim, Julan Chun, Mi-Jin Lee, Jae-Ho Shin, Hae-Jeung Lee

**Affiliations:** 1 Department of Food and Nutrition, College of BioNano Technology, Gachon University, Seongnam-si 13120, Gyeonggi-do, Republic of Korea; 2 Institute for Aging and Clinical Nutrition Research, Gachon University, Seongnam-si 13120, Gyeonggi-do, Republic of Korea; 3 Animal Welfare Research Team, National Institute of Animal Science, Rural Development Administration, Wanju 55365, Jeollabuk-do, Republic of Korea; 4 Mammidr Corporation, Seongnam-si 13524, Gyeonggi-do, Republic of Korea; 5 Department of Biomedical Laboratory Science, Eulji University, Seongnam-si 13135, Gyeonggi-do, Republic of Korea

**Keywords:** overweight, obesity, companion, mulberry leaf, anti-inflammation, *Papillibacter cinnamivorans*

## Abstract

Overweight and obesity, associated with various health complications, refer to abnormal or excessive fat accumulation conditions that harm health. Like humans, obesity is a growing problem in dogs, which may increase the risk of serious diseases such as diabetes and cancer. Mulberry leaf has shown potential anti-obesity and anti-diabetes effects in several studies. Our research studied the impact of mulberry leaf supplements in healthy old overweight dogs for 12 weeks. Blood and fecal samples were collected from the dogs before and after treatment for different analyses, including whole transcriptome and gut microbiome analysis. The Body Condition Score (BCS) and blood glucose levels were significantly decreased in all mulberry treatment groups, which justifies the anti-obesity effect of mulberry leaf in dogs. Throughout the whole transcriptome study, the downregulation of *PTX3* and upregulation of *PDCD-1*, *TNFRSF1B*, *RUNX3*, and *TICAM1* genes in the high mulberry group were found, which have been associated with anti-inflammatory effects in the literature. It may be an essential gene expression mechanism responsible for the anti-inflammatory and, subsequently, anti-obesity effects associated with mulberry leaf treatment, as confirmed by real-time polymerase chain reaction analysis. In microbiome analysis, *Papillibacter cinnamivorans*, related to the Mediterranean diet, which may cause anti-inflammatory effects, were abundant in the same treatment group. Further studies may be required to establish the gene expression mechanism and role of abundant bacteria in the anti-obesity effect of mulberry supplements in dogs. Overall, we propose mulberry leaves as a portion of food supplements for improving blood glucose levels and the anti-inflammation of blood in companion dogs.

## 1. Introduction

Obesity is an increasingly primary health concern and one of the leading causes of declining quality of life [1,2]. Obesity is strongly associated with a higher prevalence of infection and worse recovery, leading to a higher mortality rate [3,4]. Canine obesity is on the rise as much as in humans, and the prevalence of obesity in dogs is 7.6%, which continues to increase [5,6]. Obesity is considered a chronic and complex pathological state accompanied by various diseases, such as inflammation, insulin resistance, pancreatitis, and cardiovascular diseases [7]. Obesity induces low-grade systemic inflammation and affects the innate immune system’s low state of metabolic homeostasis [8,9]. Obesity-related inflammation is widely considered one of the major factors that trigger the onset of insulin resistance (IR), a major feature of type 2 diabetes [10]. In addition, insulin deficiency and hyperglycemia caused by diabetes lead to immune dysfunction in the host [11].

Food supplements are one of the preferred choices against obesity because drugs may cause serious side effects in long term usage. Mulberry (*Morus alba* L.) leaves are a nutritional supplement that has long been widely used for health purposes. Owing to its nutritional value in many Asian countries, it has been widely used as a functional food, including beverages, noodles, and herbal tea. Mulberry leaves contain numerous bioactive compounds, such as flavonoids and phenolic acids, which are responsible for their antioxidant activity. A study reported that high-dose 1-deoxynojirimycin (1-DNJ), which is found to be distributed among the mulberry species, reduces aspartate transferase (AST) and alanine transferase (ALT) levels, and increases sensitivity to fat in high-fat mice [12,13]. The 1-DNJ significantly reduces intestinal glucose absorption [14], improves the serum lipid profile in coronary heart disease [15], inhibits metastasis of melanoma cells [16], and improves insulin sensitivity [17]. In addition, the effects of mulberry leaf on both humoral and cellular immunity have been proven by increasing antibody production levels during in vitro and in vivo experiments [18,19]. Hence, we used the efficacy of these mulberry leaves to apply them to the study of diabetes and obesity through the food of old dogs. Obesity is a complex phenotype associated with numerous genes. We used blood transcriptomes to study canine aging obesity because blood transcriptomes have been found to respond to various environmental changes [20,21]. 

Diet is considered a key factor influencing the structure of the gut microbiome [22]. In recent years, the importance of the gut microbiome in obesity has been strengthened through many studies [23]. The gut microbiome sustains the body’s metabolism and energy balance. It can increase the accumulation of adipose tissue in the host [24] and contribute to obesity in different ways [25]. A gut microbiome study was also conducted as it is strongly associated with obesity and could be influenced by the mulberry leaf supplements provided in the current research. In the study of obesity, transcriptional mechanics increased our understanding of inflammation-related gene expression and began to link mRNA changes to the physiological state of obesity. In addition, mulberry leaf supplements are, for the first time, used for their anti-obesity effects in dogs. Mulberry leaf is suggested as a food supplement that may reduce obesity in dogs. Further studies are required to establish the identified gene expression for anti-inflammatory and anti-obesity effects.

## 2. Results

### 2.1. Body Condition Score (BCS) and Body Weight (BW)

**Figure 1:**
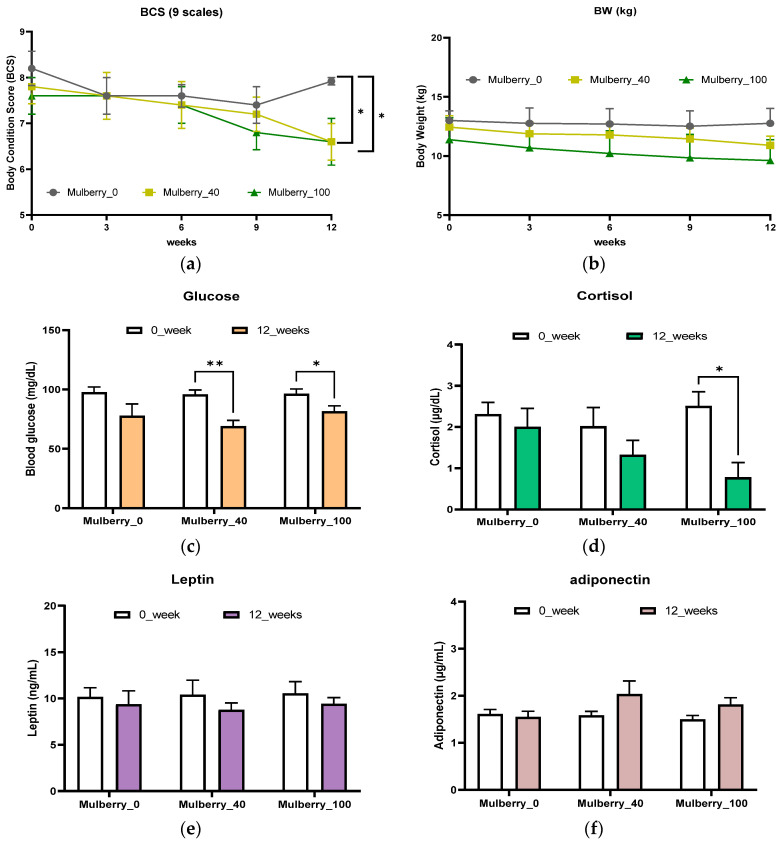
Comparison of body condition score (BCS) (**a**), body weight (BW) (**b**), glucose (**c**), cortisol (**d**), leptin (**e**), adiponectin (**f**) levels in obese dogs with low-dose (Mulberry_40) or high-dose (Mulberry_100) of mulberry leaf treatment for 12 weeks. * of BCS (**a**) shows the *p*-value of <0.05 vs. Mulberry_0 group. All data are presented as mean ± SD. * shows *p* < 0.05 and ** is *p* < 0.01. Mulberry_0: normal diet group supplemented with 40 mg/kg/day of maltodextrin (placebo); Mulberry_40: normal diet group supplemented with 40 mg/kg/day of the mulberry leaf; Mulberry_100: normal diet group supplemented with 100 mg/kg/day of the mulberry leaf.

After low- (40 mg/kg/day, Mulberry_40) or high-dose (100 mg/kg/day, Mulberry_100) mulberry leaf treatment, the BCSs of the mulberry_40 and mulberry_100 groups were significantly lower than those of the mulberry _0 group after 12 weeks (Figure 1a). The weights of the mulberry leaf treatment groups were slightly decreased for 12 weeks; however, the differences between the treatment and placebo groups were not significant (Figure 1b).

### 2.2. Obesity-Related Factors in the Blood

After 12 weeks of mulberry treatments, blood glucose levels were decreased in all mulberry treatment groups. Both the mulberry_40 and mulberry_100 groups had significantly lowered blood glucose levels, lower than that of the mulberry _0 group after 12 weeks of treatment (Figure 1c). Cortisol levels were decreased in both the mulberry_40 and mulberry_100 groups, but only showed as significantly decreased in the mulberry_100 group. Leptin and adiponectin levels were reduced and increased (respectively) in the mulberry treatment groups compared to those of the mulberry _0 group after 12 weeks, but the results were not significant (Figure 1e,f). 

### 2.3. Preprocessing and Alignment of Reads

Whole transcriptome analysis was carried out to analyze the effect of the mulberry diet on dogs using RNA-Seq analysis. The quality control step of sequencing reads achieved more than 97% (mean value > 98.8%) of good reads from every sample that passed through the filters. A high alignment percentage (mean value > 96%) of the filtered reads with the reference genome was observed (Table S1). This alignment was used for further assembly and expression analyses.

### 2.4. Assembly and Expression Analysis

**Figure 2:**
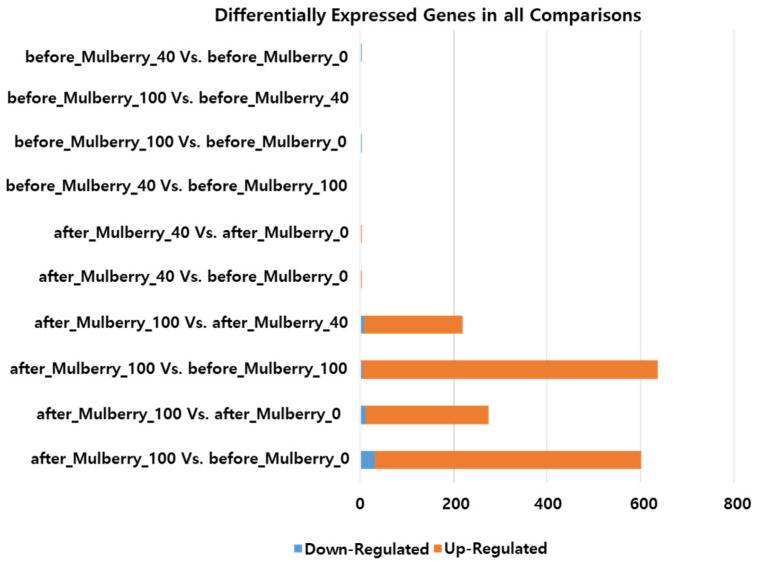
Number of differentially expressed genes before and after treatment. Mulberry_0: normal diet group supplemented with 40 mg/kg/day of maltodextrin (placebo); Mulberry_40: normal diet group supplemented with 40 mg/kg/day of the mulberry leaf; Mulberry_100: normal diet group supplemented with 100 mg/kg/day of the mulberry leaf.

Alignment was performed to assemble the whole transcriptome of all samples used in the study. Subsequently, the expression of all assembled genes/transcripts was calculated for reading counts and fragments per kilobase of transcript per million mapped reads (FPKM). Furthermore, differentially expressed genes were identified by comparison between groups. The DEGs were mainly observed when comparing the Mulberry_100 group with the others (Figure 2). According to the selected cutoff (FDR 0.1 and minimum fold change 4), a total of 601, 275, 637, and 219 DEGs were identified when the after_Mulberry_100 group was compared with before_Mulberry_0, before_Mulberry_40, before_Mulberry_100, and after_Mulberry_40, respectively (Figure 1). Graphs of the DEGs were plotted to visualize the MA and volcano plots (Figure S1).

### 2.5. Functional Enrichment Analysis and Obesity-Related Genes

Functional enrichment of significant DEGs (URGTG) revealed that most genes associated with cellular processes in the biological process category followed biological regulation (Figure S2). Congruently, most genes were enriched in the cellular and anatomical entities in the cellular component category. Most DEGs were associated with the binding and gene-specific transcriptional regulator categories in the molecular function and protein class categories. In pathway enrichment analysis, DEGs were associated with 27 diverse pathways (Figure S3). Among all common DEGs, three genes (*TNFRSF1B*, *CACNA1A*, and *AKT1S1*) were found to be associated with obesity-related pathways from the Kyoto Encyclopedia of Genes and Genomes (KEGG) pathway database.

### 2.6. Real-Time PCR Analysis for Validation

**Figure 3:**
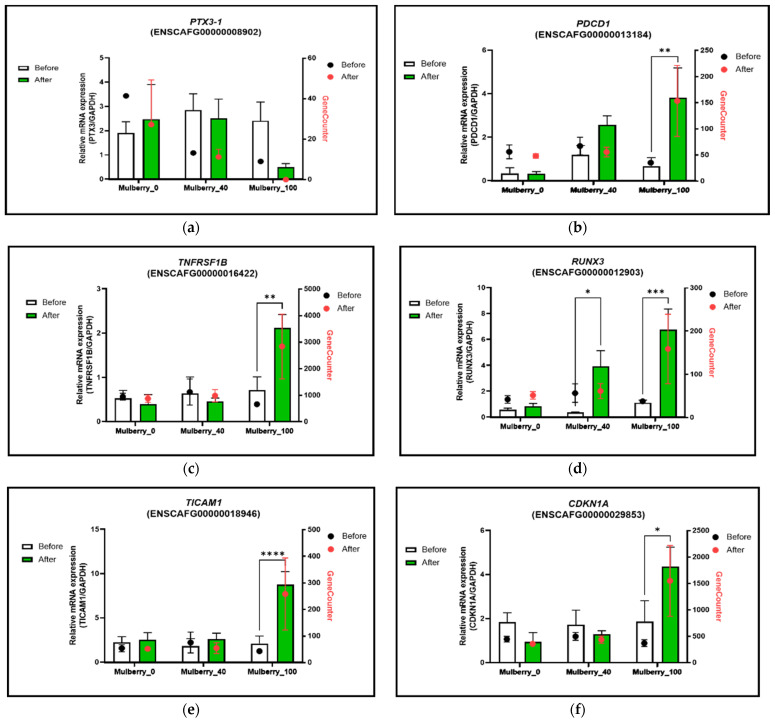
Quantitative expressions of diabetes-related genes between the before and after groups. (**a**) *PTX3*, (**b**) *PDCD1*, (**c**) *TNFRSF1B*, (**d**) *RUNX3*, (**e**) *TICAM1*, and (**f**) *CDKN1A*. All data are presented as mean ± SEM, and tests were performed in three independent experiments. * *p* < 0.05, ** *p* < 0.01, *** *p* < 0.001, and **** *p* < 0.0001. The red lines expressed GeneCounts between the before and after groups. Mulberry_0: normal diet group supplemented with 40 mg/kg/day of maltodextrin (placebo); Mulberry_40: normal diet group supplemented with 40 mg/kg/day of the mulberry leaf; Mulberry_100: normal diet group supplemented with 100 mg/kg/day of the mulberry leaf.

We conducted real-time PCR analysis to validate the upregulated and downregulated genes in mulberry leaves using total RNA from whole-blood samples from 15 old obese dogs. The Mulberry_100 group had lower gene expression levels of *PTX3* after mulberry treatment than those in the other groups. In contrast, the expression of other genes (*PDCD1, TNFRSF1B, RUNX3, TICAM1*, and *CDKN1A*) was significantly higher than that in the mulberry_0 or mulberry_40 groups after being treated in the Mulberry_100 group (Figure 3).

### 2.7. Gut Microbiome and Diversity Analysis

A total of 4,434,033 paired-end reads from the amplicon region were used for microbiome analysis. After preprocessing and filtering, 1,533,672 feature reads/operational taxonomic units (OTUs) were retained. A total of 18,164 unique features/operational taxonomic units (OTUs) were identified in all samples. 

A slight difference in alpha diversity was observed between the groups, but the difference was not significant according to the *p*-values. Similarly, the groups showed no significant differences in the evenness of alpha diversity (Figure S4). The beta diversity calculated using different methods is plotted in a 3D graph (Figure S5). In the 3D graph plotted using the Bray Curtis method, the samples with no treatment were more dispersed in the graphs than the Mulberry_100 group samples that were clustered in the middle region of the chart. The models with the Mulberry_40 group were also more dispersed in the graph than those with the Mulberry_100 group, but they were less dispersed in comparison to the Mulberry_0 group. A similar visual visualization pattern can be observed in the 3D plots generated for the other methods (Jaccard distance, unweighted UniFrac distance, and weighted UniFrac distance) (Figure S5). Again, the beta diversity difference was not significant according to the *p*-value cutoff in the permutational multivariate analysis of variance (PERMANOVA).

### 2.8. Taxonomy Analysis of the Samples

**Figure 4:**
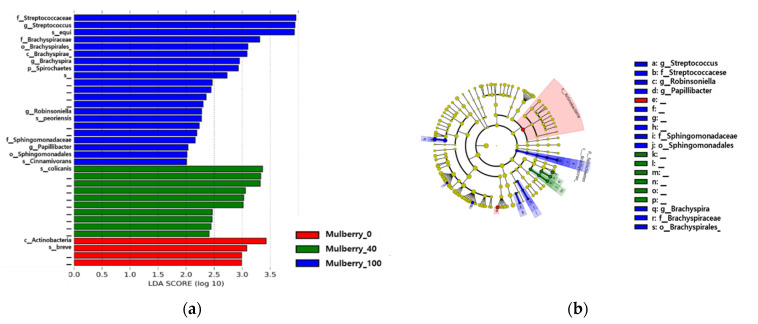
Differential abundance of taxa in the Mulberry_0, Mulberry_40, and Mulberry_100 groups. (**a**) Bar plot depicting differentially abundant bacterial taxa according to LDA. (**b**) Cladogram. Mulberry_0: normal diet group supplemented with 40 mg/kg/day of maltodextrin (placebo); Mulberry_40: normal diet group supplemented with 40 mg/kg/day of the mulberry leaf; Mulberry_100: normal diet group supplemented with 100 mg/kg/day of the mulberry leaf.

Taxonomy annotation was performed, and the results were visualized through bar plots and Krona plots to study the taxa present in the samples and groups (Figure 4). The most dominant phyla and classes present in all the models were Firmicutes and Clostridia. Other abundant phyla in most samples were Fusobacteria, Actinobacteria, and Proteobacteria (Figure 4).

### 2.9. Correlation Analysis between Obesity-Related Factors and Gut Microbiome

**Figure 5:**
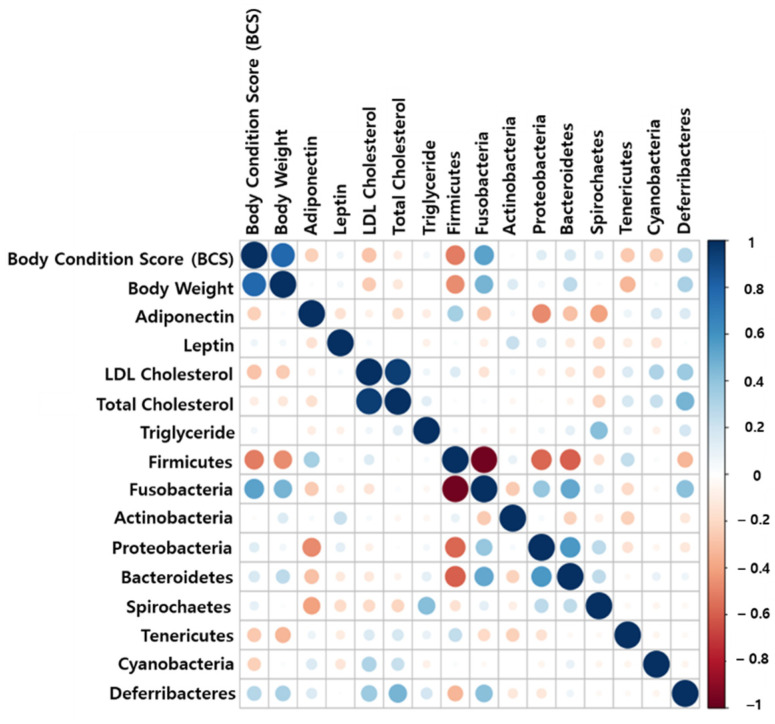
Visualization of correlation matrix among microbiome and obesity-related factors.

The study observed a weak negative correlation between phylum Firmicutes and BCS (or body weight) (Figure 5). A strong correlation was not observed between the gut microbiome and obesity-related factors. However, a strong positive correlation between BCS and body weight was detected, as expected in this study (Figure 5).

### 2.10. Functional Potential of Bacterial Community

**Figure 6:**
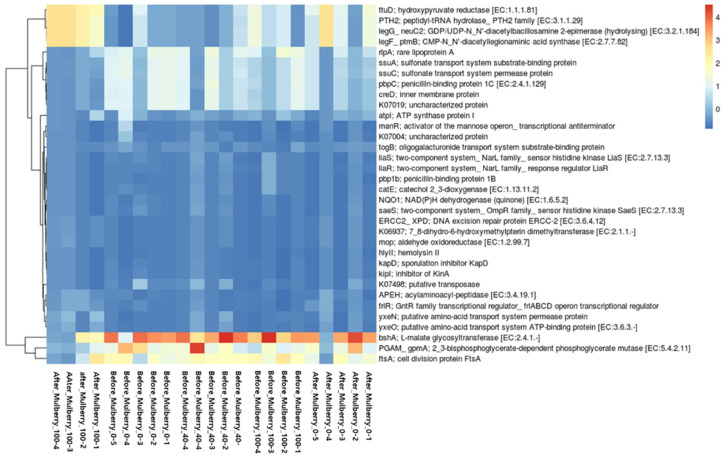
Heatmap showing the occurrence of KOs, which were differential in the comparison of the Mulberry_100 group vs. the Mulberry_0 group. Mulberry_0: normal diet group supplemented with 40 mg/kg/day of maltodextrin (placebo); Mulberry_100: normal diet group supplemented with 100 mg/kg/day of the mulberry leaf.

Enzyme classification numbers, pathways, and KEGG Ortholog (KO) abundance were analyzed using PICRUSt2 to predict the functional potential of a community based on ASVs. A total of 34, 10, and 1 KO were differentially abundant in the Mulberry_100 group vs. the Mulberry_0 group, the after_Mulberry_100 group vs. the after_Mulberry_40 group, and the after_Mulberry_0 group vs. the Mulberry_0 group (*p* < 0.05) in both Welch’s and Wilcoxon rank tests, calculated through ALDEx2 in the R platform (Tables S3 and S4). Notably, according to Benjamini–Hochberg’s corrected *p*-value, no KO was found to be differentially abundant. Furthermore, a heatmap of 34 differentially abundant KOs was drawn and visualized to compare the after_Mulberry_100 group vs. the before_Mulberry_0 group (Figure 6).

## 3. Discussion

Obesity and inflammation are important factors that may cause serious health issues in humans and other animals, such as dogs. Obesity may cause chronic inflammation, as obesity is associated with chronic inflammation in obese subjects [26], and secretion of inflammatory adipokines (such as leptin, interleukin (IL-6), tumor necrosis factor-α, monocyte chemoattractant protein-1, and resistin) are also reported to be secreted by adipocytes [27]. Weight control may reverse the expression of inflammatory markers and inflammation in obese dogs [28]. Anti-inflammation has the potential to reduce not only inflammation-related issues, but also obesity [29]. The current study was designed to consider the importance of the efficacy of mulberry leaves for the health of dogs, especially their anti-obesity [30] and anti-inflammatory effects [31,32]. 

Critically important obesity-related factors such as glucose, cortisol, leptin, and adiponectin have been studied in this study. Blood glucose levels are strongly associated with diabetes and obesity. Similarly, adiponectin has a strong role in obesity and related diseases, and its blood serum level can be decreased in obese patients [33]. Leptin is a peptide hormone that performs different functions, including the regulation of food intake and body mass [34]. Patients with abdominal obesity have elevated cortisol levels. However, not all obese individuals have higher cortisol levels [35]. In this study, we observed a decrease in blood glucose and cortisol levels after 12 weeks of oral administration in a high concentration of the Mulberry_100 group. Though there were no significant results in blood leptin and adiponectin levels in the treatment groups, a tendency to decrease or increase appeared in terms of trends. This tendency needed to be established in the genetic part.

Whole transcriptome analysis has been used to study gene expression-based mechanisms of treatment in different organisms [6,36,37,38,39]. In this study, RNA-Seq analysis was applied to study the entire transcriptome expression in the different groups of dogs to understand the biological mechanism important for the anti-inflammatory and anti-obesity effects of the diet. The whole-transcriptome analysis generated and utilized high-quality next-generation sequencing data. The quality control experiment yielded a high percentage (>98%) of high-quality reads. Furthermore, a high alignment rate (>96%) of the reads was achieved, again justifying the quality of the assembled transcriptome analyzed in this study. The standard RNA-Seq pipeline was used for differential expression analysis of different groups according to the treatments provided. Differential gene expression was mainly observed in the Mulberry_100 group, indicating that significant gene expression changes occurred only with 100 mg/kg/day mulberry leaf treatment. Therefore, the DEGs in the Mulberry_100 group were further studied. In the gene subset analysis, 143 and 2 genes were found to be upregulated and downregulated, respectively, in the Mulberry_100 group compared with all other groups. These genes may be considered potential targets for anti-obesity and/or anti-inflammatory effects of 100 mg/kg/day of mulberry leaf treatment provided to the animals. Functional enrichment analysis revealed that the majority of URGTG functions were associated with cellular processes, biological regulation, cellular anatomical entity, binding, and gene-specific transcriptional regulator categories. These categories are associated with cellular metabolic activities that can be related to inflammation and/or obesity. 

Among URGTG, three genes, *AKT1S1*, *TNFRSF1B,* and *CACNA1A*, were found to be associated with obesity-related pathways (in KEGG database), which were thermogenesis (hsa04714), the adipocytokine signaling pathway (hsa04920), and type II diabetes mellitus (hsa04930), respectively. One of these genes (*TNFRSF1B*) is also known to be important for the anti-inflammatory response. These genes may be important for the anti-obesity effect of the treatment in the current study.

Furthermore, genes from the immune system process and the interleukin signaling pathway from the biological function and pathway category were selected to explore their roles in the literature. Program cell death protein 1 (*PDCD-1*), TNF receptor superfamily member 1B (*TNFRSF1B*), Runt-related transcription factor (*RUNX3*), and TIR domain-containing adapter molecules 1 (*TICAM1*) were the four out of five genes (from the immune system process) that were found to be associated with anti-inflammatory activities. *PDCD-1* is known to modulate immune system activity by suppressing the inflammatory activity of T-cells and promoting self-tolerance [40], and was downregulated in the Mulberry_100 group. *TNFRSF1B*, along with TNF-receptor 1, forms a heterocomplex that mediates the recruitment of two anti-apoptotic proteins, c-IAP1 and c-IAP2 (ubiquitin ligases), which limit cell death and prevent inflammation [41]. *RUNX3* plays an important role in inhibiting the JAK2/STAT3 pathway, which may protect against acute lung injury and inflammation [42]. *TICAM-1* inhibits the interaction of IL-17RA with Act1 and suppresses c-Myc expression, which causes inflammation. These functions of *TICAM-1* suppress IL-17A–IL-17A–mediated inflammatory responses [43]. Similarly, the cyclin-dependent kinase inhibitor 1 (*CDKN1A*) gene from the interleukin signaling pathway may suppress inflammatory cytokine production, including IL-1β, IL-6, and TNF-α, and represent a potential therapeutic target for novel RA treatments [44]. Among the downregulated genes, pentraxin 3 (*PTX3*), one of the two *DRGTG* genes, is known as an inflammatory gene and is considered a novel biomarker for inflammatory cardiovascular disease [45]. Downregulation of *PTX3* and upregulation of *PDCD-1, TNFRSF1B, RUNX3*, *and TICAM1* may be important gene expression mechanisms in the treatment groups responsible for the anti-inflammatory effects associated with the treatment. Expression of all these candidate genes was also cross-checked through PCR in all studied animals, again following the same pattern as observed in the whole transcriptome (RNA-Seq) analysis and favoring the anti-inflammatory effect of the treatment (Figure 3).

Gut microbiome is also a crucial factor contributing to both anti-obesity and anti-inflammatory properties [25,46]. Therefore, microbiome analysis was also performed to study the gut microbiome in the studied animals, and the differential abundance of the gut microbiome in the treatment groups. Notably, sex-related differences can also exist in the gut microbiome. However, some studies have not found any difference, and the effect of sex on gut microbiomes seems to be less influential in comparison with other factors [47,48,49]. Both males and females were included in each studied group to balance the sex-related differences in the gut microbiome. In the gut microbiome analysis, both the alpha diversity index and evenness were not significantly different according to the *p*-value. Similarly, beta diversity was not significantly different among the groups according to PERMANOVA. This suggests that the treatments did not cause significant microbiome dysbiosis in the gut of the animals. Taxonomic annotation revealed that the most abundant phylum in all samples was Firmicutes, which is normally considered one of the most abundant phyla in the gut microbiome of humans and dogs [50,51]. Differential abundance analysis of the gut microbiome revealed that *Papillibacter cinnamivorans* was abundant in the Mulberry_100 groups, and other species of *Papillibacter cinnamivorans* are known to be abundant in individuals consuming a Mediterranean diet, which is known to be rich in vegetables [52]. The Mediterranean diet is known to have an anti-inflammatory effect [53], and the diet provided to the Mulberry_100 group in the current study was also rich in mulberry leaves, which could explain the abundance of *Papillibacter cinnamivorans* in this study. This study aimed to analyze the anti-inflammatory and anti-obesity effects of *P. cinnamivorans*. No strong correlation was observed between obesity-related factors and gut microbiome, which might be due to the small number of animals used in the study. Furthermore, a study with a more significant number of animals may be suggested in future research. Similarly, more studies are required to establish the identified gene expression for anti-inflammatory and subsequent anti-obesity effects. Nevertheless, Mulberry leaf is suggested as a food supplement that may reduce obesity in companion dogs.

## 4. Materials and Methods

### 4.1. Animals and Diet

The participants were elderly obese dogs visiting animal hospitals. Obese dogs identified as level six or higher out of nine levels of the body condition score (BCS) were selected through veterinary tests, and individuals with clinical symptoms, systemic diseases, or history were excluded from the test. All test subjects were included in the test after obtaining written consent from their guardians, and the test method was approved by the Animal Testing Ethics Committee (PTB-2021-IACUC-006). This study was conducted in accordance with the guidelines of the Animal Care and Use Committee of the Institute of Animal Science. Fifteen old overweight dogs (average 8.06 years, BCS 7.8 ± 0.06, and 12.36 ± 0.06 kg body weight [BW], one beagle dog, one Shetland sheepdog, and three cocker spaniels per group) were included (Table 1). A total of 15 individual dogs were randomly divided into three groups: the placebo group (Mulberry_0) with 40 mg/kg/day of maltodextrin; low concentration administered with a 40 mg/kg/day of Mulberry leaf powder; the low-dose group (Mulberry_40), and a high concentration administered with a 100 mg/kg/day of Mulberry leaf; the high-dose group (Mulberry_100). All groups were placed in capsules of the same size and color. The placebo capsule (40 mg/kg/day of maltodextrin (placebo), Mulberry_0) or test capsules (40 or 100 mg/kg/day of the mulberry leaf) were administered daily during the morning feeding time for 12 weeks according to the guidance of the veterinarian in charge. The type, feed quantity, and living environment of the feed, other than the placebo/test substance administration, remained the same as before participation in the test. Food intake was monitored during the 12 weeks of intervention through counseling over the phone at least once a week. The amount of feed and the number of snacks were measured. All dogs required four visits to the veterinary hospital for blood collection, weighing, and BCS measurements. BCS (at 3, 6, 9, and 12 weeks) measurements were performed by veterinarians using a 9-point scale based on the criteria of Laflamme et al. (1997) [54].

### 4.2. Blood Sampling, Serum Chemistry, and Fecal Sampling

Blood and fecal samples were collected from dogs before and after treatment for 12 weeks. Blood was collected and left in a tube for more than 30 min, and then centrifuged at 400× *g* at 4 °C for 10 min. The serum was stored at −80 °C until use. Canine blood glucose (Accu-Chek Mobile, Roche Diabetes Care GmbH, Mannheim, Germany), canine cortisol ELISA kit (LSBio, Inc., Seattle, WA, USA), canine leptin ELISA kit (R&D Systems, Minneapolis, MN, USA), and canine adiponectin ELISA kit (R&D Systems, Minneapolis, MN, USA) levels were analyzed using commercial kits according to the guidelines of each manufacturer. Whole blood samples were collected from dogs using RNAprotect^®^ Animal Blood Tubes (QIAGEN, Hilden, Germany) at the start of the treatment and after the end of the experiment (12 weeks) for RNA-Seq. Blood (500 μL) was purified using an RNeasy Protect Animal Blood Kit (QIAGEN, Hilden, Germany) according to the manufacturer’s instructions. Total cholesterol (TC) and triglyceride (TG) levels were analyzed using commercial kits according to the manufacturer’s protocol. Fecal samples were collected at the end of treatment and stored at −80 °C until further use.

### 4.3. RNA-Seq Pipeline Used for Assembly and Differential Expression Analysis

Quality control analysis (QCA) is the initial step of RNA-Seq, which consists of filtering, error removal, and trimming of the reads. According to standard parameters, pair-end reads from all groups were subjected to quality control analysis using fastp (version 0.23.2) [55]. Only good reads according to QCA were used for alignment-based assembly for further analysis. The reference genome of Dog (*Canis lupus familiaris*), CanFam 3.1 reference genome assembly released by the Genome Reference Consortium, was considered for the alignment of filtered (good quality) reads through HISAT2 [56]. Alignment results for each sample were obtained as sequence alignment map (SAM) files converted to sorted BAM files according to the requirement for further analysis. StringTie was used to assemble reads by aligning BAM files [57]. Assembly results of all samples were combined before analysis of the differential expression study. A Python script (prepDE.py) available with StringTie was used to connect the assembly results for all the groups. Furthermore, a matrix of reading count values for every assembled transcript/gene was obtained for differential expression analysis. A gene count matrix containing expression values for all samples was utilized for further expression-related analyses using iDEP.93 [58]. Differential gene expression analysis was performed using DESeq2 and EdgeR with a default threshold value (false discovery rate < 0.1). A fold change threshold for gene expression of 4 instead of 2 was considered to reduce the number of differentially expressed genes (DEGs).

### 4.4. Comparison of DEGs in Different Groups

Essential differentially expressed genes in the treatment group were identified by comparing gene sets found to be upregulated or downregulated between the groups using Venn analysis through InteractiVenn [59]. Common upregulated or downregulated genes in the treatment group (URGTG or DRGTG) were identified by comparing gene sets that were upregulated compared to all other groups.

### 4.5. Functional Enrichment of DEGs and Identification of Obesity-Related Genes

Functional enrichment of URGTG was performed using PANTHER (protein analysis through evolutionary relationships). URGTG was enriched according to biological processes, cellular components, molecular functions, protein classes, and pathways [60]. Ensemble IDs of all URGTG genes were used to query the panther classification system using *C. lupus familiaris* as the target organism. The results were stored in images and Microsoft Excel formats. All genes present in obesity-related pathways were collected from the KEGG pathway database. DEGs present in the obesity-related pathway were considered obesity-related genes in the analysis.

### 4.6. Blood Gene Expression Using Real-Time PCR Analysis

RNA (50 ng/μL) before and 12 weeks after treatment was isolated from the blood sample using an QIAamp RNA Blood Mini Kit (Qiagen, Hilden, Germany). Extracted RNA was used for cDNA synthesis using the iScript cDNA synthesis kit (BioRad, Hercules, CA, USA). Real-time PCR was performed using SYBR^®^ Green Master Mix (TaKaRa Bio, Otsu, Japan) and analyzed using the QuantStudio3 PCR system (Thermo Fisher Scientific, San Jose, CA, USA). The primer sequences (5′–3′) used for RT-PCR are shown in Table S2, and the expression levels were normalized to the internal GAPDH gene.

### 4.7. Gut Microbiome Sequence Analysis

16S ribosomal amplicon sequences from the V3–V4 region acquired as pair-end reads were processed using the latest version of Quantitative Insights into Microbial Ecology (QIIME2-version 2021) [61]. These paired-end reads from all groups were imported into QIIME2 and subjected to quality control before the analysis. Divisive amplicon denoising algorithm 2 (DADA2) was used to denoise, trim, remove low-quality reads, and filter chimeras [62]. Trimming was carried out after graphic visualization of forward and reverse reads for quality through the “qiime tools view module” to choose trimming locations. Amplicon sequence variants (ASVs) were constructed for further analysis. Multiple sequence alignment (MSA) of the ASVs was carried out using mafft for phylogenetic analysis [63]. FastTree was used to construct a phylogenetic tree from the alignment [64].

### 4.8. Taxonomic Annotation

A taxonomy classifier based on Greengenes 13_8 99% OTUs was used for the taxonomic annotation of ASVs using the q2-feature-classifier module in QIIME2 [65]. It uses a naïve Bayes taxonomy classifier for annotation. The taxonomic annotation of all samples was visualized using Barplots and Krona plots, which were drawn using the qiime taxa barplot” module and python script, respectively [66].

### 4.9. Diversity Analysis

Alpha diversity was calculated by measuring the community richness and evenness. Community richness was calculated using the observed features, Shannon’s diversity index, and Faith’s phylogenetic diversity. Community evenness was calculated using Pielou’s evenness method. Similarly, beta diversity was calculated through qualitative and quantitative measures of community dissimilarity using Jaccard distance and Bray-Curtis distance. Furthermore, beta diversity was calculated by incorporating a phylogenetic relationship between features through unweighted UniFrac distance (a qualitative measure of community dissimilarity). UniFrac distance (a quantitative measure of community dissimilarity) was weighted UniFrac distance (a quantitative measure of community dissimilarity).

### 4.10. Differential Abundance of Taxa

Linear discriminant analysis effect size (LEfSe) was used to identify the differential abundance of taxa in the treatment group [67]. Taxonomy results collapsed to the species level (7th level), and the ASV table results from qiime2 with metadata information were used to prepare the LEfSe input format file. Standard cutoff parameters and a one-against-all strategy for multiclass analysis were used in the LEfSe analysis. Graphs depicting differences in the microbiome community and cladograms were drawn for visualization [67].

### 4.11. Correlation Analysis between Obesity-Related Factors and Gut Microbiome

Taxonomy results collapsed to the phylum (2nd level) and species level (7th level) were used to calculate the Pearson correlation using the R program. The correlation between gut microbiomes and obesity-related factors (such as BCS, body weight, adiponectin, leptin, LDL, T-Chol, and TG) were calculated. Furthermore, the correlation matrix was plotted using the corrplot function in the R program.

### 4.12. Prediction of Functional Potential of Bacterial Community with Amplicon Sequences

Prediction of microbial community functions from amplicon sequences was carried out using PICRUSt2 (https://github.com/picrust/picrust2, accessed on 30 May 2022) in all groups. PICRSt2 was selected for the analysis, as it is considered an accurate method for predicting the functional potential of the bacterial community [68]. ASVs obtained from DADA2 were used for functional prediction as input in sequences and abundance files in the biome format through the PICRUSt2 pipeline. To identify the differential function of the microbial community in different groups, ALDeX2 was used for the predicted function in different groups.

## Figures and Tables

**Table 1:** Information on companion dog animals for each group used in the study.

Group	Species	Gender	Age (y)	BW (kg)	BCS (9-Scale)
Mulberry_0	Cocker Spaniels	F	7	11.5	8
	Beagle	F	9	16.5	9
	Cocker Spaniels	F	10	17.4	9
	Cocker Spaniels	M	10	12.1	8
	Shetland Sheepdog	F	6	11.5	7
Mulberry_40	Beagle	M	8	15.8	9
	Cocker Spaniels	M	6	11.7	8
	Cocker Spaniels	F	9	10.0	7
	Cocker Spaniels	M	9	12.9	8
	Shetland Sheepdog	F	7	11.8	7
Mulberry_100	Beagle	F	9	17.9	9
	Cocker Spaniels	M	8	12.2	8
	Shetland Sheepdog	F	6	10.8	7
	Cocker Spaniels	F	8	9.0	7
	Cocker Spaniels	F	9	11.5	7

Mulberry_0: normal diet group supplement with 40 mg/kg/day of maltodextrin(placebo); Mulberry_40: normal diet group supplement with 40 mg/kg/day of mulberry leaf; Mulberry_100: normal diet group supplement with 100 mg/kg/day of mulberry leaf.

## Data Availability

The data presented in this study are available on request from the corresponding author.

## Supplementary Materials

The following supporting information can be downloaded at: https://www.mdpi.com/article/10.3390/ijms232315215/s1.
