# Supplementary material for: Mulberry Leaf Supplements Effecting Anti-Inflammatory Genes and Improving Obesity in Elderly Overweight Dogs
Source: Int J Mol Sci. 2022 Dec 2;23(23):15215. doi: 10.3390/ijms232315215 (PMC9735752; doi:10.3390/ijms232315215)
Supplement: Supplementary file 1 [file ijms-23-15215-s001.zip › ijms-2008927-supplementary.pdf]

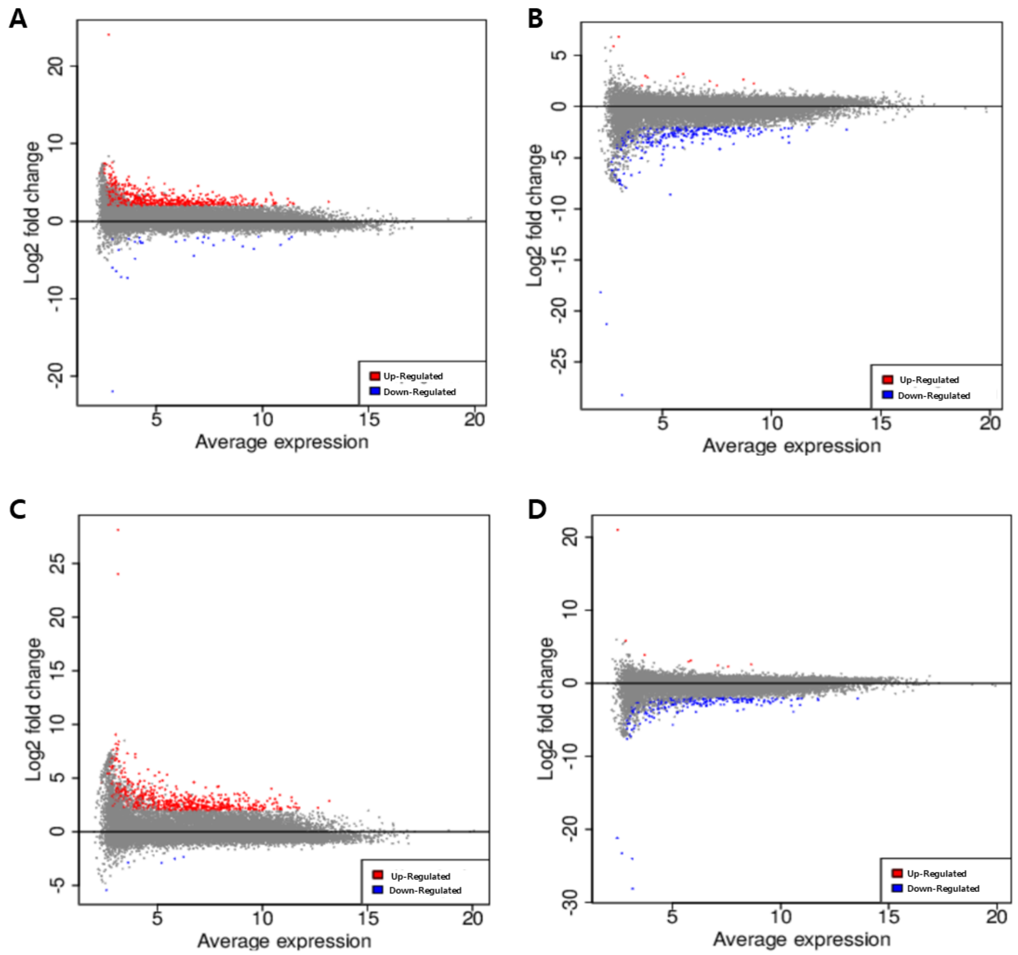

Figure S1. MA plots depicting fold change expression between the groups. (A) Comparison between After_Mulberry_100 Vs. Mulberry_0 group. (B) Comparison between After_Mulberry_100Vs. Before_Mulberry_40 group. (C) Comparison between Before_Mulberry_100 Vs. After_ Mulberry_100 group. (D) Comparison between After_Mulberry_40 Vs. After_ Mulberry_100 group. Mulberry_0: normal diet group supplement with 40 mg/kg/day of maltodextrin; Mulberry_40: normal diet group supplement with 40 mg/kg/day of mulberry leaf; Mulberry_100: normal diet group supplement with 100 mg/kg/day of mulberry leaf.

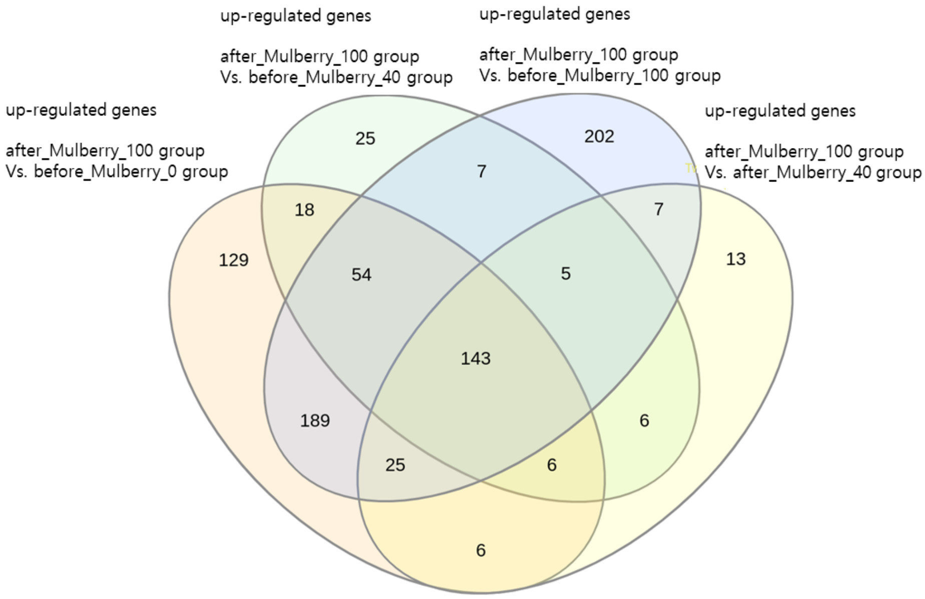

Figure S2a. Venn diagram depicting common up-regulated genes (URGTG) in the after_Mulberry_100 in comparison with before_Mulberry_0, 40, 100, and after_Mulberry_40 group.

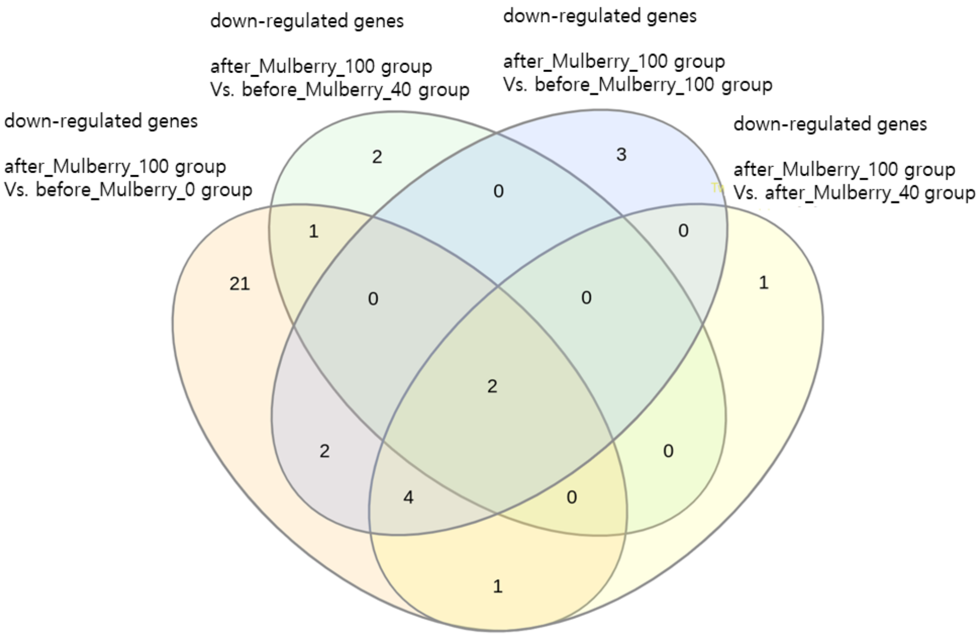

Figure S2b. Venn diagram depicting common down-regulated genes (URGTG) in the after_Mulberry_100 group in comparison with before_Mulberry_0, 40, 100, and after_Mulberry_40 group.

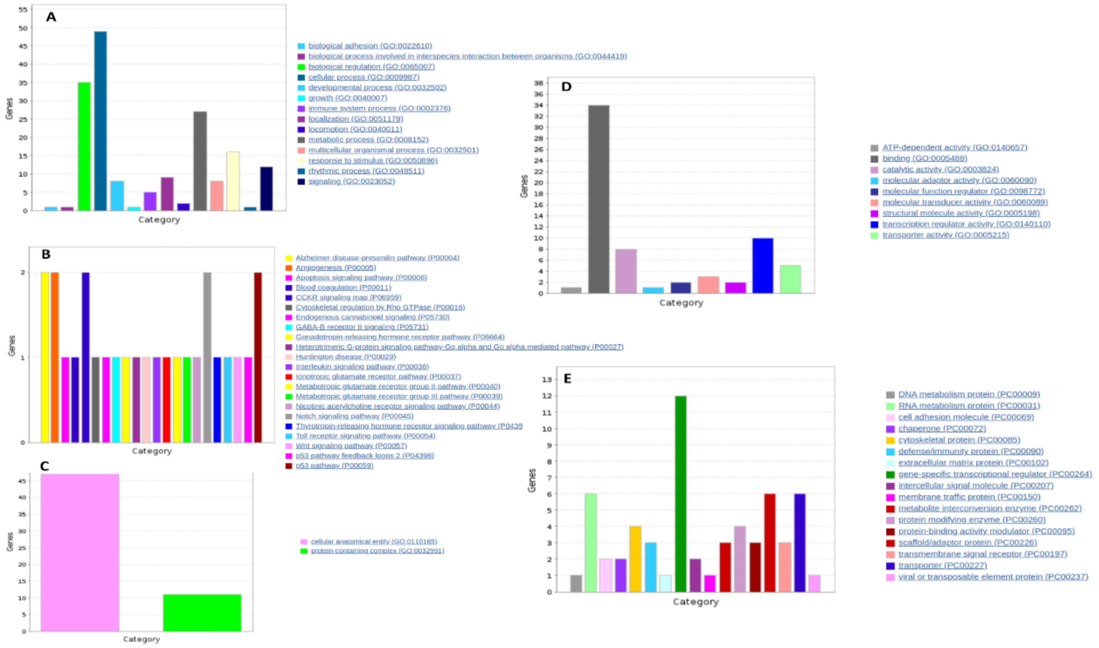

Figure S3. Functional enrichment analysis of 143 common up-regulated genes in af-ter_Mulberry_100 group (A) biological processes, (B) pathways, (C) cellular components, (D) molec-ular function, and (E) protein class

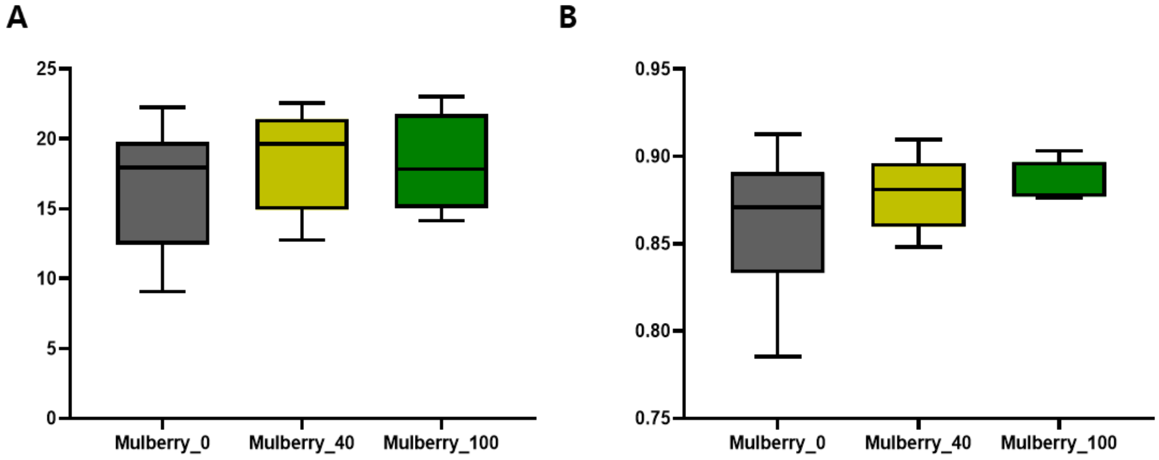

Figure S4. Alpha diversity plots of different studied groups. Faith's Phylogenetic Diversity (A) Faith's Phylogenetic Diversity. (B) Pielou's Evenness. Mulberry_0: normal diet group supplement with 40 mg/kg/day of maltodextrin; Mulberry_40: normal diet group supplement with 40 mg/kg/day of mulberry leaf; Mulberry_100: normal diet group supplement with 100 mg/kg/day of mulberry leaf.

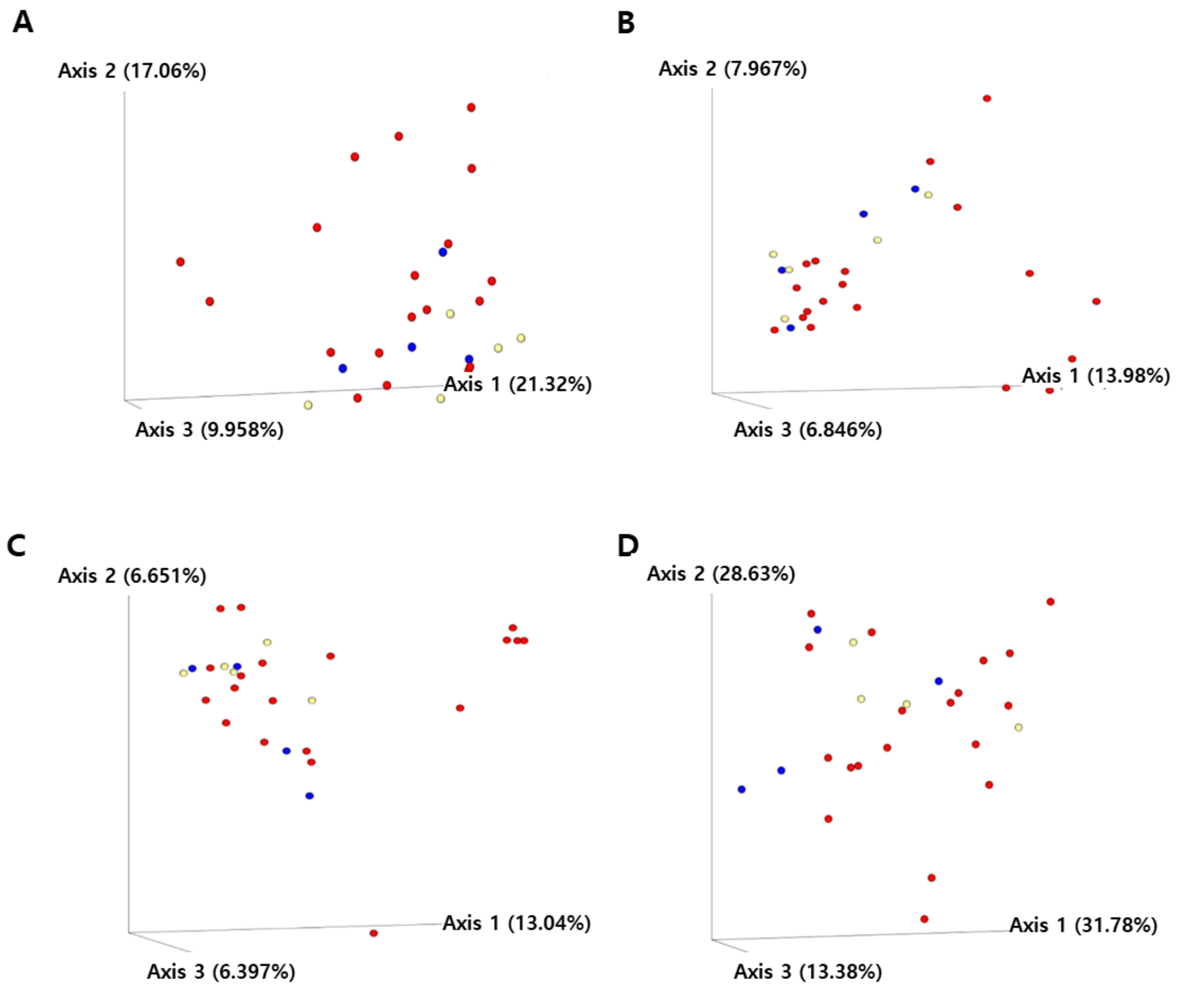

Figure S5. 3-D plots depicting Beta diversity in different groups (Red dot samples Mulberry_0 group, green dot samples from Mulberry_40 group and blue dot samples from the Mulberry_100 group). (A) 3-D plot depicting beta diversity through Bray Curtis distance. (B) 3-D plot depicting beta diversity through unweighted UniFrac distance. (C) 3-D plot depicting beta diversity through Jaccard distance. (D) 3-D plot depicting beta diversity through weighted UniFrac distance. Mulberry_0: normal diet group supplement with 40 mg/kg/day of maltodextrin; Mulberry_40: normal diet group supplement with 40 mg/kg/day of mulberry leaf; Mulberry_100: normal diet group supplement with 100 mg/kg/day of mulberry leaf.

Table S1. Details of the preprocessing and alignment results of reads from all samples.

Sample ID	# of read in million passed the filter	% reads passed the filter	Overall Alignment rate
AG1-2	81.31	99.08	97.35
AG1-3	69.05	98.62	96.59
AG1-10	73.85	98.46	96.80
AG1-11	83.26	98.40	96.76
AG1-12	65.78	98.47	97.23
AG2-1	63.64	98.31	96.96
AG2-4	76.38	98.11	98.86
AG2-5	75.28	98.82	97.15
AG2-7	77.14	98.76	97.02
AG2-13	81.48	98.74	96.61
AG3-9	73.18	98.79	96.03
AG3-14	72.77	98.69	96.60
AG3-15	61.32	97.04	78.07
AG3-16	79.24	98.54	96.54
AG3-17	86.70	98.63	96.79
BG1-2	88.41	98.90	97.18
BG1-3	75.41	99.09	97.30
BG1-10	87.27	98.13	96.78
BG1-11	87.35	98.33	96.93
BG1-12	87.81	98.35	96.88
BG2-1	79.82	98.38	96.67
BG2-4	82.76	98.46	96.94
BG2-5	84.98	98.47	96.84
BG2-7	87.27	98.37	96.32
BG2-13	58.65	98.47	96.89
BG3-9	80.11	98.85	97.12
BG3-14	72.29	98.88	97.08
BG3-15	87.76	98.69	91.55
BG3-16	80.27	98.71	97.24
BG3-17	66.67	98.73	96.94
Mean Value	77.57367	98.54233	96.134

Table S2. The primer sequences used for qPCR analysis.

Gene	Forward (5' - 3')	Reverse (5' - 3')	Size (bp)	Gene ID
PTX3	GTGGACCTGGACAACGAGAT	GGTGCCATAGGAGAACAGGA	204	ENSCAFG00000008902
PDCD1	CTACTGCTGCTGCTGACCTG	GATGGTGGCATACTCGGTCT	216	ENSCAFG00000013184
TNFRSF1B	GGAGCCAGCAAAATGACCAA	AGGTAGGAGGAGGGAGACTG	171	ENSCAFG00000016422
RUNX3	GAACGGTGGTGACTGTGATG	TGAACACGGTGATGGTCAGT	162	ENSCAFG00000012903
TICAM1	ATGCTACCAAACAGCCCAGT	CAGAGGGCAAGGACAGAGAG	163	ENSCAFG00000018946
CDKN1A	TGGGAAGACTGGCGGTATAG	GCTTGACATGTTTCCCCACT	151	ENSCAFG00000029853

Table S3. Metadata_ALDEx2.

id	Group-Dog	Gtreatment	Treatment	Gender	faith_pd
#q2:types	categorical	categorical	categorical	categorical	numeric
After_Group1-10	G1	NO	N	F	19.58920146
After_Group1-11	G1	NO	N	M	11.94270305
After_Group1-12	G1	NO	N	F	20.11770272
After_Group1-2	G1	NO	N	F	13.263933
After_Group1-3	G1	NO	N	F	19.5508649
After_Group2-1	G2	NO	N	M	20.18137904
After_Group2-13	G2	NO	N	F	19.77849671
After_Group2-4	G2	NO	N	M	12.41659131
After_Group2-5	G2	NO	N	F	9.04674898
After_Group2-7	G2	NO	N	M	18.81885452
After_Group3-14	G3	NO	N	M	15.51909631
After_Group3-16	G3	NO	N	F	11.75755856
After_Group3-17	G3	NO	N	F	12.84889792
After_Group3-9	G3	NO	N	F	22.25423952
Before_Group1-10	G1	NO	N	F	17.96645501
Before_Group1-11	G1	NO	N	M	11.45722651
Before_Group1-12	G1	NO	N	F	20.43763757
Before_Group1-2	G1	NO	N	F	18.85982563
Before_Group1-3	G1	NO	N	F	14.5619868
Before_Group2-1	G2	T	Y	M	20.25740496
Before_Group2-13	G2	T	Y	F	19.63150074
Before_Group2-4	G2	T	Y	M	17.08000515
Before_Group2-5	G2	T	Y	F	12.75442908
Before_Group2-7	G2	T	Y	M	22.57504644
Before_Group3-14	G3	T2	Y	M	23.02983887
Before_Group3-16	G3	T2	Y	F	14.1293932
Before_Group3-17	G3	T2	Y	F	17.93999444
BGBefore_Group	G3	T2	Y	F	17.70492416

Table S4. Metadata_R platform.

id	Group-Dog	Gtreatment	Treatment	Gender	pielou_evenness
#q2:types	categorical	categorical	categorical	categorical	numeric
After_Group1-10	G1	NO	N	F	0.890862181
After_Group1-11	G1	NO	N	M	0.8429761
After_Group1-12	G1	NO	N	F	0.900644018
After_Group1-2	G1	NO	N	F	0.879291184
After_Group1-3	G1	NO	N	F	0.912375425
After_Group2-1	G2	NO	N	M	0.909106859
After_Group2-13	G2	NO	N	F	0.886906924
After_Group2-4	G2	NO	N	M	0.788688076

After_Group2-5	G2	NO	N	F	0.792869899
After_Group2-7	G2	NO	N	M	0.861915507
After_Group3-14	G3	NO	N	M	0.833232544
After_Group3-16	G3	NO	N	F	0.870695762
After_Group3-17	G3	NO	N	F	0.800700007
After_Group3-9	G3	NO	N	F	0.911900814
Before_Group1-10	G1	NO	N	F	0.876725099
Before_Group1-11	G1	NO	N	M	0.785194789
Before_Group1-12	G1	NO	N	F	0.860537158
Before_Group1-2	G1	NO	N	F	0.871227178
Before_Group1-3	G1	NO	N	F	0.839615753
Before_Group2-1	G2	T	Y	M	0.871438254
Before_Group2-13	G2	T	Y	F	0.880992654
Before_Group2-4	G2	T	Y	M	0.847904359
Before_Group2-5	G2	T	Y	F	0.909467798
Before_Group2-7	G2	T	Y	M	0.882777727
Before_Group3-14	G3	T2	Y	M	0.877979742
Before_Group3-16	G3	T2	Y	F	0.878010709
Before_Group3-17	G3	T2	Y	F	0.903036717
Before_Group3-9	G3	T2	Y	F	0.876434816
